# Comparative field performance and adherence to test results of four malaria rapid diagnostic tests among febrile patients more than five years of age in Blantyre, Malawi

**DOI:** 10.1186/1475-2875-9-209

**Published:** 2010-07-20

**Authors:** Jobiba Chinkhumba, Jacek Skarbinski, Ben Chilima, Carl Campbell, Victoria Ewing, Miguel San Joaquin, John Sande, Doreen Ali, Don Mathanga

**Affiliations:** 1 Malaria Alert Centre, College of Medicine, Private Bag 360, Chichiri, Blantyre 3, Malawi; 2 Malaria Branch, Centers for Disease Control and Prevention, Atlanta, GA, USA; 3 Community Health Sciences Unit, Ministry of Health, Lilongwe, Malawi; 4 Malawi-Liverpool-Wellcome Trust, Blantyre, Malawi; 5 National Malaria Control Programme, Ministry of Health, Lilongwe, Malawi; 6 Department of Community Health, College of Medicine, Blantyre, Malawi; 7 Center for Tropical and Global Emerging Diseases, University of Georgia, Athens, Georgia, USA

## Abstract

**Background:**

Malaria rapid diagnostics tests (RDTs) can increase availability of laboratory-based diagnosis and improve the overall management of febrile patients in malaria endemic areas. In preparation to scale-up RDTs in health facilities in Malawi, an evaluation of four RDTs to help guide national-level decision-making was conducted.

**Methods:**

A cross sectional study of four histidine rich-protein-type-2- (HRP2) based RDTs at four health centres in Blantyre, Malawi, was undertaken to evaluate the sensitivity and specificity of RDTs, assess prescriber adherence to RDT test results and explore operational issues regarding RDT implementation. Three RDTs were evaluated in only one health centre each and one RDT was evaluated in two health centres. Light microscopy in a reference laboratory was used as the gold standard.

**Results:**

A total of 2,576 patients were included in the analysis. All of the RDTs tested had relatively high sensitivity for detecting any parasitaemia [Bioline SD (97%), First response malaria (92%), Paracheck (91%), ICT diagnostics (90%)], but low specificity [Bioline SD (39%), First response malaria (42%), Paracheck (68%), ICT diagnostics (54%)]. Specificity was significantly lower in patients who self-treated with an anti-malarial in the previous two weeks (odds ratio (OR) 0.5; p-value < 0.001), patients 5-15 years old versus patients > 15 years old (OR 0.4, p-value < 0.001) and when the RDT was performed by a community health worker versus a laboratory technician (OR 0.4; p-value < 0.001). Health workers correctly prescribed anti-malarials for patients with positive RDT results, but ignored negative RDT results with 58% of patients with a negative RDT result treated with an anti-malarial.

**Conclusions:**

The results of this evaluation, combined with other published data and global recommendations, have been used to select RDTs for national scale-up. In addition, the study identified some key issues that need to be further delineated: the low field specificity of RDTs, variable RDT performance by different cadres of health workers and the need for a robust quality assurance system. Close monitoring of RDT scale-up will be needed to ensure that RDTs truly improve malaria case management.

## Background

In response to increasing levels of resistance to conventional monotherapies, such as chloroquine, amodiaquine and sulphadoxine-pyrimethamine, most countries in sub-Saharan Africa have introduced artemisinin-based combination therapy (ACT) for the treatment of uncomplicated malaria [1]. The change in drug policy, combined with aggressive vector control, coincides with a decrease in malaria transmission and subsequent decline in the proportion of fevers attributable to malaria [2]. Although the targeting of anti-malarials only to patients who need them has always been important, changes in drug policy and malaria epidemiology have increased the need for laboratory-based diagnosis of malaria as a means to prevent the emergence of ACT resistance and improve overall clinical management of febrile patients [3,4].

The World Health Organization (WHO) now recommends malaria case management based on parasite-based diagnosis in all cases [5]. Given the difficulty with implementing microscopy-based definitive diagnosis of malaria, malaria rapid diagnostic tests (RDTs) have been suggested as an alternative [6,7]. In line with WHO recommendations, the Malawi National Malaria Control Programme (NMCP) has developed new malaria treatment algorithms, which incorporate the use of RDTs for definitive diagnosis of malaria in patients aged ≥5 years who seek care in health facilities where malaria microscopy is not available.

In preparation for nationwide implementation of RDTs, the NMCP reviewed the commercially available RDTs regarding their target antigen, sensitivity, specificity, shelf-life, heat stability, cost and reliability of suppliers to choose an RDT for national use. Although data is available to inform this selection, much of it comes from field evaluations of a single product at a time with few studies providing head-to-head comparisons of more than one product under operational settings [8-13]. Since factors may vary across regions and countries, the Malawi NMCP requested locally-generated data to aid with the selection of an RDT for national use. Thus, four commercially available RDTs that were being considered as potential candidates for nationwide implementation were evaluated. The study objectives were to assess field sensitivity and specificity, identify factors that might affect RDT field performance, assess prescriber adherence to RDT test results, and explore operational issues regarding RDT implementation.

## Methods

### Study site

The study was conducted in Blantyre District between January and April 2009. Blantyre is located in the southern region of Malawi and has a population of 999,491 persons, the majority of whom reside in Blantyre city. Malaria is a major public health problem in the district with 506,029 clinically diagnosed malaria cases treated in public health facilities in 2009, of which 57% were aged five or more years [14]. Transmission of malaria in the district is stable throughout the year with a peak in the rainy season (November to March). More than 90% of malaria infections are caused by *Plasmodium falciparum*. Access to malaria interventions in the district is low with 43% of households owning an insecticide-treated mosquito net, and only 22% of children < 5 years of age, who had a fever in the previous two weeks receive treatment with an appropriate anti-malarial [15].

The district has one referral hospital and 22 public health centres. In Blantyre city, there is a private health sector with four large private hospitals and about 30 private clinics. This study was conducted in one urban (< 10 kilometers from the city centre; Bangwe), one peri-urban (< 20 kilometres from the city centre; Chileka) and two rural (≥20 km from the city centre; Lilangwe and Mdeka) public health centres. The health centres were selected based on the outpatient volume (> 200 per day) and availability of clinical staff and microscopy. Two health facilities, Bangwe and Chileka, had microscopy for malaria diagnosis. Health centres are staffed by different cadres of health workers: clinical officers, medical assistants, laboratory technicians, nurses, and community health workers. Community health workers in Malawi, known as Health Surveillance Assistants (HSAs), are a salaried cadre in the health system that undergo six weeks of pre-service training. They provide preventive and clinical services in the community as well as assist with the delivery of preventive and clinical services at health centres. Given the shortage of laboratory technicians in Malawi, community health workers have been proposed to perform RDTs in health facilities without laboratory technicians.

### Study design

A cross-sectional study design was used to assess the performance of four histidine rich-protein-type-2 (HRP2) based RDTs: Paracheck-Pf Device (Orchid Biomedical Systems, India; Catalogue number 30301025), ICT Malaria Pf Cassette Test (ICT Diagnostics, South Africa), SD Bioline Malaria Antigen P.f (Standard Diagnostics, Korea; Catalogue number 05FK50) and First Response Malaria (HRP2) Antigen (Premier Medical, India; Catalogue number I13FRC30). Each RDT was evaluated at only one health centre except Paracheck-Pf, which was evaluated at two health centres.

### Study procedures

#### Training of health workers

All health workers underwent cadre-specific training. Clinicians and nurses had one-day refresher training on malaria diagnosis and treatment based on the new national malaria treatment guidelines. During this training, the diagnosis and treatment of malaria patients using an algorithm which included the use of RDTs was reviewed. In sites with microscopy, health workers were instructed to treat patients according to local microscopy results. In the two sites without microscopy, health workers were instructed to treat patients according to RDT results.

Laboratory technicians from the two health facilities with microscopy underwent refresher training on malaria microscopy. Community health workers from the facilities without microscopy services received three-day training on blood sample collection via a finger prick and preparation of malaria thick slides. Both laboratory technicians and community health workers received training on how to use all four RDTs according to manufacturer's instructions. In addition, job aids that contained information on criteria for study eligibility, RDT-based treatment algorithm, instructions on how to prepare and interpret RDTs according to a modified version of the generic WHO job aid were provided to all health workers [16].

#### Enrollment of participants, data collection and patient protection

Eligible participants were consecutively enrolled in the outpatient departments of the selected health centres. Inclusion criteria were age ≥5 years, a documented fever or a history of fever in the previous 24 hours and consent to participate in the study. Pregnant women and patients with severe illness were excluded from the study.

**Figure 1:**
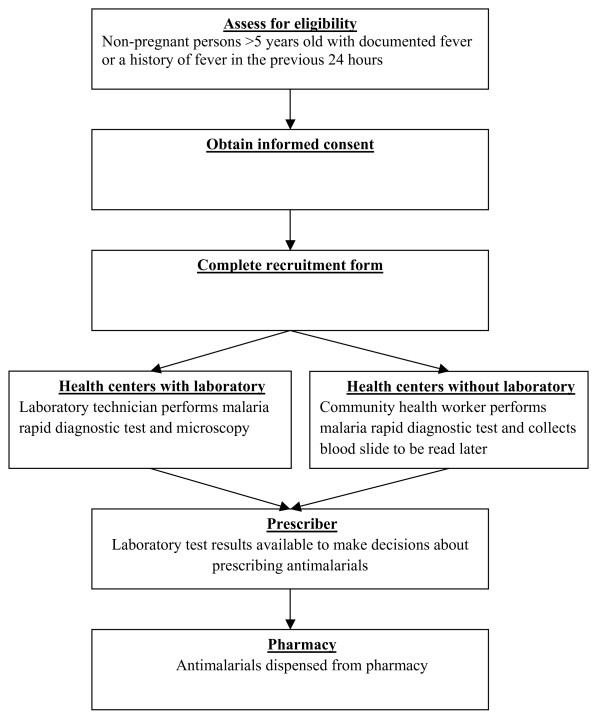
**Flow of patients in malaria rapid diagnostic test study**.

Clinicians and nurses enrolled study participants and collected demographic and clinical information using a structured form. Completed forms were collected once weekly from the study sites. At the time of collection, forms were checked for completeness and consistency of recorded information and where possible, missing data and discrepancies were corrected on site in consultation with the health worker who had completed the form. Patient flow through the study is presented in Figure 1.

#### Malaria diagnosis

An RDT was conducted on all enrolled patients using whole blood collected via a finger prick. The reading and interpretation of results were based on the specific instructions provided by the RDT manufacturer in the package insert. Thick malaria smears were prepared from the same finger prick blood sample and air-dried by laboratory staff. In the two health centres with microscopy, the slides were stained with Field's Stain A and B (azure dye and eosin) by the local laboratory technician and read locally before being sent to a central laboratory at the College of Medicine once a week. Slides from the two health centres without microscopy were sent directly to the central laboratory where they were stained with Field's Stain A and B by an expert microscopist. At the central laboratory all slides were independently read by two expert microscopists. Discordant results on presence or absence of parasitaemia between the two expert microscopists were resolved by referring to a third expert microscopist. All laboratory staff reading microscopy slides both locally and at the central laboratory were blinded to RDT and other microscopy results. A blood slide was considered negative if no trophozoites were seen after examining 100 high power fields (100 × objective)[17]. Malaria parasite count per microlitre of blood from the thick film was estimated by multiplying the average number of parasites per high power field by 500 [17]. Gametocytes were not counted.

### Sample size and power

To estimate 90% RDT sensitivity compared to expert microscopy with 80% power and 95% confidence interval of ± 5%, 138 patients with parasitaemia by expert microscopy were required [18]. Assuming a slide positivity rate of 40% in patients more than five years of age presenting with fever and adjusting for at least 10% for refusals we set out to recruit a minimum of 384 febrile patients per RDT.

### Data analysis

The primary outcome measures were RDT sensitivity and specificity using microscopy as a gold standard. In addition, we assessed prescriber adherence to RDT test results. Data was double entered in SPSS version 12.0 (SPSS Inc., Chicago, Illinois, USA) and analyzed using STATA version 10 (StataCorp, College Station, Texas, USA) and SAS version 9.2 (SAS Institute, Cary, NC, USA). Chi square statistics were used to compare differences between persons tested with the different RDTs. Due to confounding between RDT type and health centre characteristics, such as the cadre of health worker who performed the RDT, two different logistic regression models were used to assess the effects of RDT type and RDT operator (e.g. cadre of health worker performing the RDT) on RDT sensitivity and specificity. Model 1 assessed the variables: RDT type, measured temperature (≥37.5°C vs. < 37.5°C), geometric mean parasite density (≥5000 asexual parasites per microlitre vs. < 5000 asexual parasites per microlitre), and history of self-treatment with anti-malarials in the last two weeks. Model 2 assessed the variables: measured temperature (≥37.5°C vs. < 37.5°C), geometric mean parasite density (≥5000 asexual parasites per microlitre vs. < 5000 asexual parasites per microlitre), history of self-treatment with anti-malarials in the last two weeks, and cadre of health worker performing the RDT (community health worker versus laboratory technician). An assessment of RDT type and RDT operator could not be done in a unified multivariable logistic regression model due to confounding.

### Ethical approval

This study was approved by the Malawi College of Medicine ethical committee and the United States Centers for Disease Control and Prevention Institutional Review Board.

## Results

A total of 2,679 patients were recruited, but only 2,573 (96%) patients with complete RDT and expert microscopy results were included in the analysis. Demographic characteristics varied significantly between persons tested with the different RDTs (Table 1). Malaria prevalence varied significantly at the different RDT testing sites (p < 0.001); the prevalence of microscopically confirmed malaria increased from urban to semi-urban and to rural settings: 3%, 16% and 39% respectively. Overall reported self-treatment with anti-malarials was 12%, but this varied significantly among the different RDT testing sites with a range of 5% in Lilangwe and 29% in Chileka (p < 0.001).

**Table 1:** Health facility and patient characteristics in malaria rapid diagnostic test (RDT) evaluation in Blantyre, Malawi, 2009

RDT name	Bioline SD	First response malaria	ICT diagnostics	Paracheck		Total	P value*
**Health centre name**	**Mdeka (N = 476)**	**Lilangwe (N = 508)**	**Chileka (N = 683)**	**Bangwe (N = 573)**	**Mdeka (N = 333)**	**(N = 2,573)**	

Health facility characteristics							
Location	Rural	Rural	Semi-urban	Urban	Rural		
Person performing RDT	CHW^‡^	CHW^‡^	Laboratory Technician	Laboratory Technician	CHW^‡^		
Patient characteristics							
Female	59%	60%	59%	56%	65%	59%	0.13
Child 5-15 years old	44%	56%	37%	36%	30%	42%	< 0.001
Owns a mosquito net Used a mosquito net the previous night	54% 50%	62% 63%	51% 48%	43% 41%	58% 61%	53% 52%	< 0.001 < 0.001
Self treated with antimalarial in past 2 weeks	8%	5%	29%	8%	2%	12%	< 0.001
Presumptive malaria diagnosis	100%	99%	27%	92%	100%	89%	< 0.001
Local microscopy positive^†^	na	na	69%	29%	na	51%	< 0.001
RDT positive	76%	72%	53%	25%	66%	56%	< 0.001
Expert microscopy positive	41%	40%	16%	3%	34%	25%	< 0.001

* Statistical testing using chi square test for trend

^† ^Local microscopy available only in Chileka and Bangwe Health Centres

^‡ ^CHW = Community health worker

### Sensitivity and specificity of malaria rapid diagnostic tests

SD Bioline Malaria Antigen P.f had the highest sensitivity at 97% followed by First Response Malaria (HRP2) Antigen at 92% (Table 2). The overall sensitivity of Paracheck-Pf was 90% but this varied by site with a sensitivity of 96% (185/201) in Mdeka and only 59% (10/17) in Bangwe with the Bangwe results based on relatively few samples. Using a logistic regression model adjusting for age group (5-15 years vs. > 15 years), measured temperature (≥ 37.5°C vs. < 37.5°C), geometric mean parasite density (≥ 5,000 asexual parasites per microlitre vs. < 5,000 asexual parasites per microlitre), and history of self treatment with anti-malarials in the last two weeks, none of the RDTs tested were significantly more sensitive than ICT Malaria Pf (Table 2).

**Table 2:** Predictors of malaria rapid diagnostic test (RDT) sensitivity (RDT positive, expert microscopy positive) in Blantyre, Malawi, 2009

	Sensitivity n/N(%)	Unadjusted odds ratio (P value)	Adjusted odd ratio Model 1 (P value)	Adjusted odds ratio Model 2 (P value)
RDT type				
Bioline SD	188/193 (97)	2.6 (0.17)	2.9 (0.14)	
First response malaria	185/201 (92)	1.1 (0.82)	1.1 (0.84)	
Paracheck	118/130 (91)	0.8 (0.63)	0.8 (0.77)	
ICT diagnostics	98/109 (90)	Referent	Referent	
Age				
5 to ≤ 15 yrs	369/382 (97)	4.8 (< 0.001)	5.1 (< 0.001)	5.1 (< 0.001)
> 15 yrs	206/237 (87)	Referent	Referent	Referent
Temperature				
≥ 37.5°C	281/298 (94)	2.1 (0.09)	1.7 (0.23)	1.7 (0.22)
< 37.5°C	286/310 (92)	Referent	Referent	Referent
Geometric mean parasite density				
≥ 5000 per μl	181/193 (94)	1.1 (0.74)	0.7 (0.36)	0.6 (0.32)
< 5000 per μl	408/440 (93)	Referent	Referent	Referent
Self-treated with antimalarial in past 2 weeks				
Yes	44/51 (86)	0.9 (0.85)	1.2 (0.82)	2.1 (0.38)
No	501/538 (93)	Referent	Referent	Referent
RDT performed by:				
Community health worker	481/507 (95)	2.6 (0.03)		3.1 (0.02)
Laboratory technician	108/126 (86)	Referent		Referent

Model 1 assesses RDT type adjusted for patient age, temperature, parasite density, and self-treatment with anti-malarials.

Model 2 assesses RDT test operator (community health worker versus laboratory technician) adjusted for patient age, temperature, parasite density, and self-treatment with anti-malarials.

Overall, there were 44/633 (7%) false negative RDT results (RDT negative, gold standard microscopy positive). Of the 44 patients that had false negative RDT results, four (9%) had an asexual parasite density of < 200 parasites per microlitre, 4 (9%) had an asexual parasite density of 200-499 parasites per microlitre, 10 (23%) had a parasite density of 500-5,000 parasites per microlitre and 26 (59%) had a density of > 5,000 parasites per microlitre.

All of the RDTs tested had low specificity ranging from 39% for SD Bioline Malaria Antigen P.f to 68% for Paracheck-Pf (Table 3). Using a logistic regression model adjusting for age group (5-15 years vs. > 15 years), measured temperature (≥37.5°C vs. < 37.5°C), and history of self-treatment with anti-malarials in the last two weeks, SD Bioline Malaria Antigen P.f and First Response Malaria (HRP2) Antigen had significantly lower specificity than ICT Malaria P.f, while Paracheck-Pf had significantly higher specificity than ICT Malaria P.f (Table 3).

**Table 3:** Predictors of malaria rapid diagnostic test (RDT) specificity (RDT negative, expert microscopy negative) in Blantyre, Malawi, 2009

	Specificity n/N(%)	Unadjusted odds ratio (P value)	Adjusted odd ratio Model 1 (P value)	Adjusted odds ratio Model 2 (P value)
RDT type				
Bioline SD	109/283 (39)	0.5 (< 0.001)	0.5 (< 0.001)	
First response malaria	128/307 (42)	0.6 (< 0.001)	0.7 (0.01)	
Paracheck	530/776 (68)	1.8 (< 0.001)	2.1 (< 0.001)	
ICT diagnostics	311/574 (54)	Referent	Referent	
Age				
5 to ≤ 15 yrs	278/680 (41)	0.4 (< 0.001)	0.4 (< 0.001)	0.4 (< 0.001)
> 15 yrs	778/1211 (64)	Referent	Referent	Referent
Temperature				
≥37.5°C	422/790 (53)	0.8 (0.03)	0.8 (0.02)	0.9 (0.20)
< 37.5°C	640/1092 (59)	Referent	Referent	Referent
Self-treated with antimalarial in past 2 weeks				
Yes	126/254 (50)	0.7 (0.02)	0.7 (0.05)	0.5 (< 0.001)
No	920/1605 (57)	Referent	Referent	Referent
RDT performed by:				
Community health worker	346/810 (43)	0.4 (< 0.001)		0.4 (< 0.001)
Laboratory technician	732/1130 (65)	Referent		Referent

Model 1 assesses RDT type adjusted for patient age, temperature and self-treatment with anti-malarials.

Model 2 assesses RDT test operator (community health worker versus laboratory technician) RDT test operator adjusted for patient age, temperature and self-treatment with anti-malarials.

In comparison to the field performance of RDTs, the sensitivity and specificity of local microscopy were 88% (15/17) and 74% (401/544) in Bangwe, 91% (99/109) and 36% (204/574) in Chileka, 91% (114/126) and 54% (605/1128) in both sites combined, respectively.

### Predictors of malaria rapid diagnostic test performance

Given the relatively high sensitivity, but coupled with low specificity of all RDTs tested, potential factors for this phenomenon were explored. Given the design of this study where any RDT was only performed by either a community health worker or a laboratory technician, it was not possible to ascertain whether the measured sensitivity and specificity was due to the properties of the test (RDT itself) or the test operator (community health worker versus laboratory technician). However, using a logistic regression model adjusting for age group (5-15 years vs. > 15 years), measured temperature (≥37.5°C vs. < 37.5°C), and history of self-treatment with anti-malarials in the last two weeks, it was found that for all RDTs the sensitivity was significantly higher (p = 0.02) and the specificity significantly lower (p < 0.001) when the RDTs were performed by community health workers compared to laboratory technicians (Tables 2 and 3).

In addition, the sensitivity was significantly higher and the specificity significantly lower in patients 5-15 years old compared to patients > 15 years, even after adjusting for other potential confounders such as RDT type, measured temperature (≥37.5°C vs. 37.5°C), and history of self-treatment with anti-malarials in the last two weeks. Lastly, history of self-treatment with anti-malarials in the last two weeks was associated with lower specificity (Table 3). Geometric parasite density was not found to be significantly associated with RDT sensitivity in either the univariate or multivariate models.

### Health worker adherence to malaria diagnostic test results and training instructions

Health workers adhered to microscopy and RDT positive results and prescribed anti-malarials for over 98% of patients who had positive test results (Table 4). Of note, health workers prescribed anti-malarials for almost all patients with discordant results (microscopy positive and RDT negative) according to training. However, health workers rarely withheld treatment, even in the setting of negative test results; 58% of patients with a negative RDT result were still treated with an anti-malarial despite our training. Only 7% of patients who were both microscopy-negative and RDT-negative were prescribed an anti-malarial.

**Table 4:** Health worker adherence to malaria diagnostic test results and training instructions in Blantyre, Malawi, 2009

Test results	Training instructions	Treated with anti-malarial n/N (%)
Sites with local microscopy		
Microscopy positive, RDT positive	Treat for malaria	366/368 (99)
Microscopy positive, RDT negative	Treat for malaria	283/286 (99)
Microscopy negative, RDT negative	Do not treat for malaria	34/471 (7)
Microscopy negative, RDT positive	Do not treat for malaria	83/155 (54)
Sites without local microscopy		
RDT positive	Treat for malaria	985/1,005 (98)
RDT negative	Do not treat for malaria	223/385 (58)

RDT = Malaria rapid diagnostic test

## Discussion

Decreasing malaria transmission leading to a decline in the proportion of fevers attributable to malaria in sub-Saharan Africa and the use of relatively expensive first-line anti-malarials have increased the importance of accurate malaria diagnosis at all levels of the health system [2]. Malaria RDTs must have both high (> 95%) sensitivity and specificity in field settings. High sensitivity is necessary to ensure that true cases of malaria are detected and appropriately managed while high specificity is needed to avoid false positive results that would lead not only to unnecessary anti-malarial treatment but also a missed diagnosis of the true cause of non-malarial fever.

The sensitivity of the RDTs evaluated in this study are similar to the results of other published studies [8-12]. Surprisingly, all of the RDTs evaluated had relatively low specificity resulting in high false positive rates compared to most published studies [8-13]. Only a few studies have reported low specificity [19,20]. Both biological and operational factors that could have resulted in low specificity in this study were explored. HRP2 is known to persist in the blood stream for several weeks and some loss of specificity might be due to patients with circulating antigens, but not live parasites that would be detected by microscopy [20]. In this study, 12% of patients had self-treated with anti-malarials prior to presenting for care to the health facility and might have cleared their parasitaemia, but have residual circulating antigens. The analysis shows that even after adjusting for other factors, self-treatment was significantly associated with lower specificity.

However, even after adjusting for self-treatment with anti-malarials in the past two weeks, two other factors that do not have a plausible biological explanation were independently associated with low specificity: being a patient 5-15 years of age and having the test performed by a community health worker. It is unclear why specificity was lower in patients 5-15 years of age. However, it is possible that health workers interpreting RDTs were more likely to err on the side of caution and interpret questionable test results as positive resulting in reduced specificity. In particular, the specificity was significantly lower when the RDT was performed by a community health worker compared to a laboratory technician. This demonstrates that despite their relative ease of use, the accuracy of RDT results is affected by the cadre of the person performing them. This finding has important implications for training and decisions about which cadre of staff are selected for performing the tests. Access to malaria diagnosis in rural areas is essential for promoting rational clinical care and community health workers have been shown to reliably perform RDTs in other settings [21]. However it is vital that health workers base their clinical decisions on accurate diagnostic test results. Training, certification and supervision of community health workers on RDT use, should therefore be rigorous enough to ensure the reliability and validity RDT test results in field settings. In addition, the specificity is also relatively low (54%) for routine microscopy at the health facilities in this study. The low specificity of both RDTs and microscopy is concerning because it can potentially misguide clinicians, compromise the cost effectiveness of RDTs, and might contribute to the emergence of drug resistance [22-25]. More research needs to be done to further explore this phenomenon, especially in light of upcoming plans to implement RDTs in health facilities in Malawi. This evaluation highlights critical areas for operational research, including who should perform RDTs and how to monitor RDT performance in health facilities.

Over-diagnosis of malaria, involving provision of anti-malarial drugs to patients without evidence of parasitaemia is well documented, and this study documents the magnitude of this problem in Blantyre, Malawi [26-30]. In Malawi, for a considerable period of time, all fevers have been assumed to be malaria. Health education messages have stressed the importance of treating malaria and few diagnostic facilities have been available to support other diagnoses. It is unsurprising, therefore, that over half of all negative test results in this study were disregarded. Nonetheless, this observation serves to highlight that roll out of RDTs should be accompanied by comprehensive package for health workers. This should include not only in-service training emphasizing the need to target malaria treatment to patients with laboratory-confirmed parasitaemia, but also provide alternative treatment for patients with negative malaria diagnostics tests results who have another cause for their febrile illness. Improvement of overall management of febrile patients in remote areas will have to promote use of diagnostic tools for both malaria and other febrile illnesses [31]. Although difficult to interpret since most health workers in routine practice will not have access to both microscopy and RDT results, health workers rarely treated patients with anti-malarials when both microscopy and RDT were negative. Further research into why health workers disregard malaria diagnostic test results is needed given the substantial resources that will be put into RDT scale-up.

## Limitations

Different cadres of health care workers (clinical officers, medical assistants, nurses, community health workers) were involved in the study for both patient recruitment and treatment. The staff mix in the study sites was not uniform. In general health workers in facilities with microscopy were higher ranking than those in remote facilities without microscopy. Abilities to competently manage febrile patients in the sites were, therefore, inherently different. This may have affected degree of adherence to study procedures, RDT manufacturer's instructions, the quality of information collected and ultimately the performance of the RDT tests. In particular, a key limitation of the study was the confounding of RDT type and the health worker cadre who was performing the test (community health worker versus laboratory technician). Although, it was attempted to assess the importance of these two factors on RDT performance by using different multivariable models, it was not possible to fully assess the effects of the test versus the test operator in a single unified model.

Although this was a pilot study used to help guide policy development in Malawi regarding the choice of RDT, other factors such as heat stability, lot-to-lot variation, and ease of use, which would influence the eventual choice of RDT for national policy were not tested. Neither was an evaluation of other practical considerations for country programmes, such as the relative cost of different RDTs and manufacturing capacity of suppliers carried out. Although over 2,500 patients were assessed, the results of this study are meant to be supplementary to other more detailed evaluations, such as the World Health Organization report entitled "Malaria Rapid Diagnostic Test Performance Results of WHO product testing of malaria RDTs: Round 2 (2009)" [6,7].

## Conclusions

This evaluation was designed to provide local data for decision making by the Malawi National Malaria Control Programme regarding the scale-up of RDTs in Malawi. The results of this evaluation, combined with other published data and the World Health Organization product testing of malaria RDTs, have been used to help select RDTs for national scale-up [6]. This pilot exercise has highlighted some key issues that need further exploration as Malawi proceeds with RDT scale-up. The low specificity of both RDTs and routine microscopy needs to be further delineated. In addition, operational research on strategies to monitor RDT performance and develop a robust quality assurance system is needed. Lastly, the question of variable RDT performance by different cadres of health workers has to be addressed. Malawi is embarking on rapid scale-up of RDTs in health facilities with the goal of universal diagnosis of all persons more than five years of age. Close monitoring will be needed to ensure that RDTs truly improve malaria case management.

## Competing interests

The authors declare that they have no competing interests.

## Authors' contributions

JC participated in the design of the study, supervised data collection, participated in data analysis and drafted the manuscript. JS performed the statistical analysis and reviewed drafted manuscript. BM participated in study design and supervised laboratory work. CC participated in study design. VE supervised data collection and supported drafting of manuscript. MS supported drafting of both analysis plan and the manuscript. JS and DA participated in health worker training, supervised data collection and reviewed manuscript. DM designed the study and supported drafting of manuscript. All authors read and approved the final manuscript.
